# (*E*)-*N*′-(3-Nitro­benzyl­idene)-4-(8-quinol­yloxy)butano­hydrazide

**DOI:** 10.1107/S1600536810022257

**Published:** 2010-06-16

**Authors:** Li Zeng, Xiao-Si Zhou, Li-Hua Lv, Geng-Xi Li

**Affiliations:** a Department of Pharmacy, Shaoyang Medical College, Shaoyang, Hunan 422000, People’s Republic of China

## Abstract

In the title Schiff base compound, C_20_H_18_N_4_O_4_, the conformation along the bond sequence linking the benzene and quinoline rings is *trans–*(+)*gauche–trans–trans–*(+)*gauche–trans–trans*. The dihedral angle between the aromatic ring systems is 80.3 (6)°. In the crystal, a pair of inter­molecular N—H⋯N hydrogen bonds link the mol­ecules into centrosymmetric *R*_2_^2^(20) dimers, which are aggregated *via* π–π inter­actions into sheets [quinoline–benzene ring centroid–centroid separation = 3.572 (2)–3.773 (3) Å].

## Related literature

For a closely related isomeric structure and background references, see: XiaHou *et al.* (2010 ▶). For further synthetic details, see: Zheng *et al.* (2006 ▶). For reference bond lengths, see: Allen *et al.* (1987 ▶). For hydrogen-bond motifs, see: Bernstein *et al.* (1995 ▶).
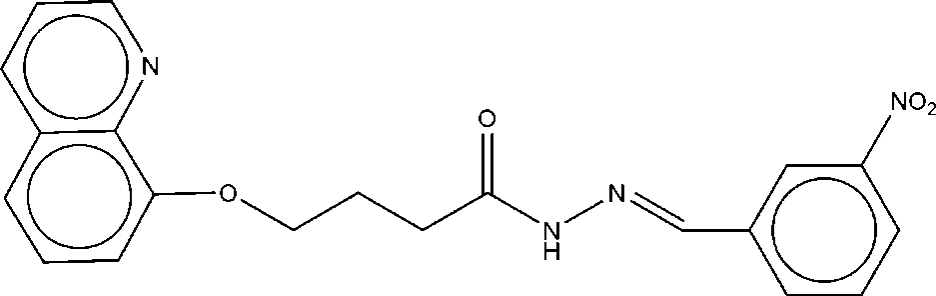


## Experimental

### 

#### Crystal data

C_20_H_18_N_4_O_4_
*M* *_r_* = 378.38
Triclinic, 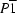

*a* = 8.3664 (12) Å
*b* = 10.4882 (15) Å
*c* = 11.5855 (16) Å
α = 100.595 (3)°
β = 91.968 (3)°
γ = 101.898 (4)°
*V* = 975.0 (2) Å^3^
*Z* = 2
Mo *K*α radiation
μ = 0.09 mm^−1^
*T* = 296 K
0.19 × 0.17 × 0.15 mm

#### Data collection

Bruker SMART CCD diffractometer
Absorption correction: multi-scan (*SADABS*; Sheldrick, 1996 ▶) *T*_min_ = 0.983, *T*_max_ = 0.986
5434 measured reflections
3409 independent reflections
1926 reflections with *I* > 2σ(*I*)
*R*_int_ = 0.023

#### Refinement

*R*[*F* ^2^ > 2σ(*F* ^2^)] = 0.065
*wR*(*F* ^2^) = 0.268
*S* = 1.08
3409 reflections
253 parameters
H-atom parameters constrained
Δρ_max_ = 0.45 e Å^−3^
Δρ_min_ = −0.26 e Å^−3^

### 

Data collection: *SMART* (Bruker, 2007 ▶); cell refinement: *SAINT* (Bruker, 2007 ▶); data reduction: *SAINT*; program(s) used to solve structure: *SHELXS97* (Sheldrick, 2008 ▶); program(s) used to refine structure: *SHELXL97* (Sheldrick, 2008 ▶); molecular graphics: *SHELXTL* (Sheldrick, 2008 ▶); software used to prepare material for publication: *SHELXTL*.

## Supplementary Material

Crystal structure: contains datablocks global, I. DOI: 10.1107/S1600536810022257/hb5488sup1.cif

Structure factors: contains datablocks I. DOI: 10.1107/S1600536810022257/hb5488Isup2.hkl

Additional supplementary materials:  crystallographic information; 3D view; checkCIF report

## Figures and Tables

**Table 1:** Hydrogen-bond geometry (Å, °)

*D*—H⋯*A*	*D*—H	H⋯*A*	*D*⋯*A*	*D*—H⋯*A*
N2—H2*A*⋯N1^i^	0.86	2.19	3.022 (4)	162

Symmetry code: (i) 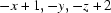.

**Fig. 1.**
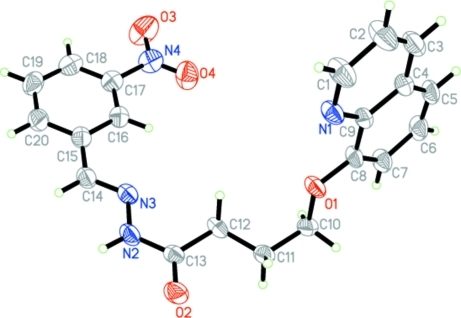
The molecular structure of (I), with displacement ellipsoids at the 30% probability level.

**Fig. 2.**
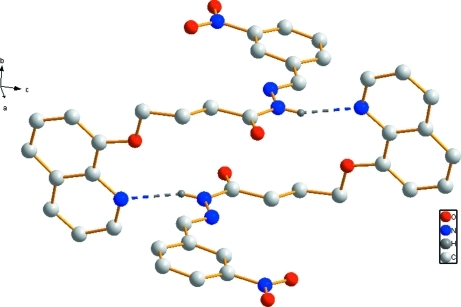
The cyclic hydrogen-bonded dimer in (I) with hydrogen bonds shown as dashed lines. H atoms, except for those involved in hydrogen bonds, are not included.

**Fig. 3.**
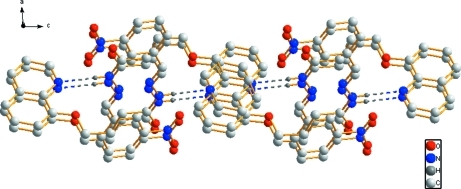
Part of the crystal structure of (I) showing hydrogen bonds as dashed lines. H atoms, except for those involved in hydrogen bonds, are not included.

## supplementary crystallographic information

### Comment

In this article, we present the synthesis and crystal structure of a new Schiff base, (I), which contains oxygen and nitrogen donors and flexible aliphatic spacers.   A closely related structure with the nitro group at the 4-position was reported recently (XiaHou *et al.*, 2010). X-ray diffraction analysis reveals that (I) contains a *trans-*(+)*gauche-trans-trans-*(+)*gauche-trans-trans* conformation along the quinoline ring–benzene ring bond sequence [torsion angles (°): C8–O1–C10–C11, 178.5 (3); O1–C10–C11–C12, 70.1 (4); C10–C11–C12–C13, -173.2 (3); C11–C12–C13–N2, -174.8 (3); C12–C13–N2–N3, 0.8 (5); C13–N2–N3–C14, -176.8 (3); N2–N3–C14–C15, -180.0 (3)] (Fig.1). The bond lengths and angles in (I) are in good agreement with the expected values (Allen *et al.*, 1987). The C14—N3 and C13—O2 bond length of 1.276 (5) and 1.214 (4) Å, respectively, indicate the presence of a typical C═N and C═O. The C═N–N angle of 116.6 (3) ° is significantly smaller than the ideal value of 120 ° expected for *sp*^2^-hybridized N atoms. This is probably a consequence of repulsion between the nitrogen lone pairs and the adjacent N atom (Zheng *et al.*, 2006). In the crystal structure, a pair of intermolecular N—H···N hydrogen bonds link the molecules into centrosymmetric cyclic *R*^2^_2_(20) (Bernstein *et al.*, 1995) dimers(Fig.2) which are aggregated *via*π–π interactions into parallel sheets [quinoline–benzene ring centroid separation = 3.572 (2)–3.773 (3) Å], giving a supramolecular two dimensional network(Fig. 3).

### Experimental

The title compound was synthesized according to the method of Zheng *et al.* (2006): 4-(quinolin-8-yloxy)butanehydrazide (0.01 mol), 3-nitrobenzaldehyde (0.01 mol), ethanol (40 ml) and some drops of acetic acid were added to a 100 ml flask and refluxed for 6 h. After cooling to room temperature, the solid product was separated by filtration. Yellow blocks of (I) were obtained by slow evaporation of a tetrahydrofuran solution of the title compound over a period of six days.

### Refinement

All H atoms were placed in idealized positions (C—H = 0.93–0.97 Å, N—H = 0.86 Å and refined as riding atoms with *U*_iso_(H) = 1.2*U*_eq_(C or N).

### Crystal data

**Table:**

C_20_H_18_N_4_O_4_	*Z* = 2
*M**_r_* = 378.38	*F*(000) = 396
Triclinic, *P*1	*D*_x_ = 1.289 Mg m^−^^3^
Hall symbol: -P 1	Mo *K*α radiation, λ = 0.71073 Å
*a* = 8.3664 (12) Å	Cell parameters from 1258 reflections
*b* = 10.4882 (15) Å	θ = 2.4–24.1°
*c* = 11.5855 (16) Å	µ = 0.09 mm^−^^1^
α = 100.595 (3)°	*T* = 296 K
β = 91.968 (3)°	Block, yellow
γ = 101.898 (4)°	0.19 × 0.17 × 0.15 mm
*V* = 975.0 (2) Å^3^	

### Data collection

**Table:**

Bruker SMART CCD diffractometer	3409 independent reflections
Radiation source: fine-focus sealed tube	1926 reflections with *I* > 2σ(*I*)
graphite	*R*_int_ = 0.023
φ and ω scans	θ_max_ = 25.1°, θ_min_ = 1.8°
Absorption correction: multi-scan (*SADABS*; Sheldrick, 1996)	*h* = −9→9
*T*_min_ = 0.983, *T*_max_ = 0.986	*k* = −12→11
5434 measured reflections	*l* = −13→12

### Refinement

**Table:**

Refinement on *F*^2^	Primary atom site location: structure-invariant direct methods
Least-squares matrix: full	Secondary atom site location: difference Fourier map
*R*[*F*^2^ > 2σ(*F*^2^)] = 0.065	Hydrogen site location: inferred from neighbouring sites
*wR*(*F*^2^) = 0.268	H-atom parameters constrained
*S* = 1.08	*w* = 1/[σ^2^(*F*_o_^2^) + (0.1425*P*)^2^ + 0.1889*P*] where *P* = (*F*_o_^2^ + 2*F*_c_^2^)/3
3409 reflections	(Δ/σ)_max_ < 0.001
253 parameters	Δρ_max_ = 0.45 e Å^−^^3^
0 restraints	Δρ_min_ = −0.26 e Å^−^^3^

### Special details

**Table:**

Geometry. All e.s.d.'s (except the e.s.d. in the dihedral angle between two l.s. planes) are estimated using the full covariance matrix. The cell e.s.d.'s are taken into account individually in the estimation of e.s.d.'s in distances, angles and torsion angles; correlations between e.s.d.'s in cell parameters are only used when they are defined by crystal symmetry. An approximate (isotropic) treatment of cell e.s.d.'s is used for estimating e.s.d.'s involving l.s. planes.
Refinement. Refinement of *F*^2^ against ALL reflections. The weighted *R*-factor *wR* and goodness of fit *S* are based on *F*^2^, conventional *R*-factors *R* are based on *F*, with *F* set to zero for negative *F*^2^. The threshold expression of *F*^2^ > σ(*F*^2^) is used only for calculating *R*-factors(gt) *etc*. and is not relevant to the choice of reflections for refinement. *R*-factors based on *F*^2^ are statistically about twice as large as those based on *F*, and *R*- factors based on ALL data will be even larger.

### Fractional atomic coordinates and isotropic or equivalent isotropic displacement parameters (Å^2^)

**Table:**

	*x*	*y*	*z*	*U*_iso_*/*U*_eq_	
O1	0.2886 (3)	−0.1013 (3)	0.68540 (19)	0.0613 (8)	
O2	0.2305 (4)	−0.0360 (3)	1.0959 (2)	0.0740 (9)	
O3	0.8883 (6)	0.6809 (5)	0.8111 (4)	0.1341 (17)	
O4	0.6888 (6)	0.5092 (4)	0.7739 (3)	0.1053 (12)	
N1	0.5334 (4)	−0.2125 (3)	0.6100 (2)	0.0625 (9)	
N2	0.4230 (4)	0.1503 (3)	1.1241 (3)	0.0642 (9)	
H2A	0.4367	0.1491	1.1978	0.077*	
N3	0.5149 (4)	0.2515 (3)	1.0792 (3)	0.0566 (8)	
C1	0.6550 (6)	−0.2666 (5)	0.5723 (4)	0.0872 (15)	
H1	0.7215	−0.2894	0.6277	0.105*	
C2	0.6912 (7)	−0.2923 (6)	0.4538 (4)	0.0991 (17)	
H2	0.7791	−0.3306	0.4316	0.119*	
C3	0.5935 (6)	−0.2595 (5)	0.3724 (4)	0.0786 (13)	
H3	0.6141	−0.2765	0.2933	0.094*	
C4	0.4625 (5)	−0.2005 (4)	0.4064 (3)	0.0592 (10)	
C5	0.3618 (6)	−0.1590 (4)	0.3276 (3)	0.0685 (12)	
H5	0.3790	−0.1723	0.2478	0.082*	
C6	0.2416 (6)	−0.1004 (4)	0.3671 (3)	0.0711 (12)	
H6	0.1778	−0.0709	0.3147	0.085*	
C7	0.2099 (5)	−0.0826 (4)	0.4869 (3)	0.0670 (11)	
H7	0.1221	−0.0456	0.5119	0.080*	
C8	0.3055 (5)	−0.1187 (4)	0.5660 (3)	0.0558 (10)	
C9	0.4356 (5)	−0.1781 (4)	0.5281 (3)	0.0524 (9)	
C10	0.1507 (5)	−0.0514 (5)	0.7282 (3)	0.0621 (10)	
H10A	0.0496	−0.1098	0.6910	0.074*	
H10B	0.1552	0.0363	0.7112	0.074*	
C11	0.1582 (5)	−0.0454 (4)	0.8588 (3)	0.0617 (11)	
H11A	0.0553	−0.0295	0.8878	0.074*	
H11B	0.1703	−0.1309	0.8740	0.074*	
C12	0.2951 (5)	0.0599 (4)	0.9259 (3)	0.0560 (10)	
H12A	0.2762	0.1465	0.9191	0.067*	
H12B	0.3971	0.0506	0.8913	0.067*	
C13	0.3110 (5)	0.0523 (4)	1.0542 (3)	0.0507 (9)	
C14	0.6227 (5)	0.3359 (4)	1.1503 (3)	0.0624 (10)	
H14	0.6351	0.3263	1.2282	0.075*	
C15	0.7263 (5)	0.4469 (4)	1.1114 (3)	0.0629 (11)	
C16	0.7079 (5)	0.4681 (4)	0.9964 (3)	0.0597 (10)	
H16	0.6269	0.4117	0.9428	0.072*	
C17	0.8108 (5)	0.5730 (4)	0.9636 (4)	0.0637 (11)	
C18	0.9289 (6)	0.6604 (4)	1.0400 (5)	0.0755 (13)	
H18	0.9950	0.7321	1.0160	0.091*	
C19	0.9478 (6)	0.6398 (5)	1.1533 (5)	0.0840 (14)	
H19	1.0292	0.6964	1.2064	0.101*	
C20	0.8451 (6)	0.5343 (5)	1.1875 (4)	0.0761 (13)	
H20	0.8571	0.5224	1.2646	0.091*	
N4	0.7921 (6)	0.5887 (4)	0.8403 (4)	0.0813 (11)	

### Atomic displacement parameters (Å^2^)

**Table:**

	*U*^11^	*U*^22^	*U*^33^	*U*^12^	*U*^13^	*U*^23^
O1	0.0655 (17)	0.0903 (19)	0.0358 (12)	0.0323 (15)	0.0088 (11)	0.0135 (12)
O2	0.077 (2)	0.095 (2)	0.0570 (16)	0.0159 (17)	0.0094 (14)	0.0357 (16)
O3	0.118 (3)	0.132 (3)	0.157 (4)	−0.016 (3)	−0.020 (3)	0.094 (3)
O4	0.135 (3)	0.091 (2)	0.082 (2)	−0.001 (2)	−0.010 (2)	0.030 (2)
N1	0.069 (2)	0.087 (2)	0.0414 (16)	0.0367 (19)	0.0070 (15)	0.0150 (16)
N2	0.076 (2)	0.081 (2)	0.0409 (16)	0.020 (2)	0.0077 (16)	0.0199 (16)
N3	0.062 (2)	0.068 (2)	0.0473 (17)	0.0246 (17)	0.0096 (15)	0.0160 (16)
C1	0.105 (4)	0.119 (4)	0.056 (2)	0.064 (3)	0.011 (2)	0.020 (2)
C2	0.114 (4)	0.135 (5)	0.065 (3)	0.070 (4)	0.018 (3)	0.010 (3)
C3	0.082 (3)	0.106 (3)	0.047 (2)	0.031 (3)	0.011 (2)	0.001 (2)
C4	0.064 (3)	0.071 (3)	0.0405 (19)	0.011 (2)	0.0023 (17)	0.0085 (17)
C5	0.087 (3)	0.078 (3)	0.0318 (18)	0.002 (2)	−0.0028 (19)	0.0081 (18)
C6	0.081 (3)	0.086 (3)	0.044 (2)	0.013 (3)	−0.013 (2)	0.013 (2)
C7	0.065 (3)	0.092 (3)	0.047 (2)	0.029 (2)	−0.0074 (18)	0.009 (2)
C8	0.067 (3)	0.067 (2)	0.0328 (17)	0.017 (2)	−0.0001 (16)	0.0066 (16)
C9	0.057 (2)	0.065 (2)	0.0346 (17)	0.0117 (19)	0.0013 (15)	0.0093 (16)
C10	0.046 (2)	0.085 (3)	0.055 (2)	0.013 (2)	0.0090 (17)	0.013 (2)
C11	0.049 (2)	0.090 (3)	0.050 (2)	0.020 (2)	0.0113 (17)	0.014 (2)
C12	0.067 (3)	0.072 (2)	0.0379 (18)	0.032 (2)	0.0137 (17)	0.0140 (17)
C13	0.046 (2)	0.067 (2)	0.047 (2)	0.0224 (19)	0.0096 (17)	0.0211 (19)
C14	0.064 (3)	0.079 (3)	0.049 (2)	0.024 (2)	0.0043 (19)	0.012 (2)
C15	0.060 (3)	0.073 (3)	0.059 (2)	0.030 (2)	0.0037 (19)	0.002 (2)
C16	0.064 (3)	0.056 (2)	0.061 (2)	0.024 (2)	0.0003 (19)	0.0053 (18)
C17	0.064 (3)	0.057 (2)	0.078 (3)	0.030 (2)	0.006 (2)	0.014 (2)
C18	0.066 (3)	0.054 (3)	0.104 (4)	0.018 (2)	0.011 (3)	0.000 (2)
C19	0.066 (3)	0.082 (3)	0.088 (4)	0.016 (3)	−0.005 (2)	−0.021 (3)
C20	0.072 (3)	0.083 (3)	0.065 (3)	0.019 (3)	0.005 (2)	−0.007 (2)
N4	0.087 (3)	0.070 (3)	0.097 (3)	0.022 (2)	0.005 (2)	0.035 (2)

### Geometric parameters (Å, °)

**Table:**

O1—C8	1.378 (4)	C7—H7	0.9300
O1—C10	1.429 (4)	C8—C9	1.406 (5)
O2—C13	1.214 (4)	C10—C11	1.501 (5)
O3—N4	1.231 (5)	C10—H10A	0.9700
O4—N4	1.207 (5)	C10—H10B	0.9700
N1—C1	1.313 (5)	C11—C12	1.495 (5)
N1—C9	1.377 (4)	C11—H11A	0.9700
N2—C13	1.355 (5)	C11—H11B	0.9700
N2—N3	1.372 (4)	C12—C13	1.505 (5)
N2—H2A	0.8600	C12—H12A	0.9700
N3—C14	1.276 (5)	C12—H12B	0.9700
C1—C2	1.406 (6)	C14—C15	1.454 (6)
C1—H1	0.9300	C14—H14	0.9300
C2—C3	1.363 (6)	C15—C20	1.368 (6)
C2—H2	0.9300	C15—C16	1.399 (5)
C3—C4	1.399 (6)	C16—C17	1.372 (5)
C3—H3	0.9300	C16—H16	0.9300
C4—C5	1.407 (5)	C17—C18	1.367 (6)
C4—C9	1.419 (5)	C17—N4	1.474 (6)
C5—C6	1.334 (6)	C18—C19	1.379 (7)
C5—H5	0.9300	C18—H18	0.9300
C6—C7	1.408 (5)	C19—C20	1.382 (6)
C6—H6	0.9300	C19—H19	0.9300
C7—C8	1.350 (5)	C20—H20	0.9300
			
C8—O1—C10	117.5 (3)	C12—C11—C10	114.0 (3)
C1—N1—C9	117.9 (3)	C12—C11—H11A	108.8
C13—N2—N3	120.9 (3)	C10—C11—H11A	108.8
C13—N2—H2A	119.5	C12—C11—H11B	108.8
N3—N2—H2A	119.5	C10—C11—H11B	108.8
C14—N3—N2	116.6 (3)	H11A—C11—H11B	107.7
N1—C1—C2	124.3 (4)	C11—C12—C13	112.4 (3)
N1—C1—H1	117.9	C11—C12—H12A	109.1
C2—C1—H1	117.9	C13—C12—H12A	109.1
C3—C2—C1	118.0 (4)	C11—C12—H12B	109.1
C3—C2—H2	121.0	C13—C12—H12B	109.1
C1—C2—H2	121.0	H12A—C12—H12B	107.9
C2—C3—C4	120.8 (4)	O2—C13—N2	119.8 (3)
C2—C3—H3	119.6	O2—C13—C12	123.5 (4)
C4—C3—H3	119.6	N2—C13—C12	116.6 (3)
C3—C4—C5	123.5 (3)	N3—C14—C15	120.9 (4)
C3—C4—C9	117.2 (3)	N3—C14—H14	119.6
C5—C4—C9	119.2 (4)	C15—C14—H14	119.6
C6—C5—C4	120.1 (3)	C20—C15—C16	118.1 (4)
C6—C5—H5	119.9	C20—C15—C14	120.4 (4)
C4—C5—H5	119.9	C16—C15—C14	121.5 (4)
C5—C6—C7	121.0 (4)	C17—C16—C15	119.2 (4)
C5—C6—H6	119.5	C17—C16—H16	120.4
C7—C6—H6	119.5	C15—C16—H16	120.4
C8—C7—C6	120.8 (4)	C18—C17—C16	122.5 (4)
C8—C7—H7	119.6	C18—C17—N4	119.9 (4)
C6—C7—H7	119.6	C16—C17—N4	117.6 (4)
C7—C8—O1	125.3 (3)	C17—C18—C19	118.5 (5)
C7—C8—C9	119.8 (3)	C17—C18—H18	120.7
O1—C8—C9	114.9 (3)	C19—C18—H18	120.7
N1—C9—C8	119.1 (3)	C18—C19—C20	119.6 (4)
N1—C9—C4	121.9 (3)	C18—C19—H19	120.2
C8—C9—C4	119.0 (3)	C20—C19—H19	120.2
O1—C10—C11	107.0 (3)	C15—C20—C19	122.1 (4)
O1—C10—H10A	110.3	C15—C20—H20	118.9
C11—C10—H10A	110.3	C19—C20—H20	118.9
O1—C10—H10B	110.3	O4—N4—O3	124.2 (4)
C11—C10—H10B	110.3	O4—N4—C17	118.8 (4)
H10A—C10—H10B	108.6	O3—N4—C17	116.9 (5)
			
C13—N2—N3—C14	−176.8 (3)	C8—O1—C10—C11	178.5 (3)
C9—N1—C1—C2	−0.4 (8)	O1—C10—C11—C12	70.1 (4)
N1—C1—C2—C3	−0.3 (9)	C10—C11—C12—C13	−173.2 (3)
C1—C2—C3—C4	0.8 (8)	N3—N2—C13—O2	−179.7 (3)
C2—C3—C4—C5	177.1 (5)	N3—N2—C13—C12	0.8 (5)
C2—C3—C4—C9	−0.7 (7)	C11—C12—C13—O2	5.7 (5)
C3—C4—C5—C6	−178.3 (4)	C11—C12—C13—N2	−174.8 (3)
C9—C4—C5—C6	−0.7 (6)	N2—N3—C14—C15	−180.0 (3)
C4—C5—C6—C7	−1.9 (7)	N3—C14—C15—C20	−177.9 (3)
C5—C6—C7—C8	3.2 (7)	N3—C14—C15—C16	2.4 (5)
C6—C7—C8—O1	177.6 (4)	C20—C15—C16—C17	1.4 (5)
C6—C7—C8—C9	−1.8 (6)	C14—C15—C16—C17	−178.9 (3)
C10—O1—C8—C7	5.6 (6)	C15—C16—C17—C18	−1.7 (5)
C10—O1—C8—C9	−175.0 (3)	C15—C16—C17—N4	177.5 (3)
C1—N1—C9—C8	−179.3 (4)	C16—C17—C18—C19	1.8 (6)
C1—N1—C9—C4	0.5 (6)	N4—C17—C18—C19	−177.3 (4)
C7—C8—C9—N1	179.0 (4)	C17—C18—C19—C20	−1.7 (6)
O1—C8—C9—N1	−0.3 (5)	C16—C15—C20—C19	−1.3 (6)
C7—C8—C9—C4	−0.8 (6)	C14—C15—C20—C19	178.9 (4)
O1—C8—C9—C4	179.8 (3)	C18—C19—C20—C15	1.5 (6)
C3—C4—C9—N1	0.0 (6)	C18—C17—N4—O4	178.7 (4)
C5—C4—C9—N1	−177.8 (4)	C16—C17—N4—O4	−0.4 (6)
C3—C4—C9—C8	179.8 (4)	C18—C17—N4—O3	1.3 (6)
C5—C4—C9—C8	2.0 (6)	C16—C17—N4—O3	−177.9 (4)

### Hydrogen-bond geometry (Å, °)

**Table:**

*D*—H···*A*	*D*—H	H···*A*	*D*···*A*	*D*—H···*A*
N2—H2A···N1^i^	0.86	2.19	3.022 (4)	162

Symmetry codes: (i) −*x*+1, −*y*, −*z*+2.
